# Studying the effects of several heat-inactivated bacteria on colon and breast cancer cells

**DOI:** 10.22099/mbrc.2019.33958.1413

**Published:** 2019-06

**Authors:** Parisa Rabiei, Hassan Mohabatkar, Mohabatkar Behbahani

**Affiliations:** Department of Biotechnology, Faculty of Advanced Sciences and Technologies, University of Isfahan, Isfahan, 81746-73441, Iran

**Keywords:** Inflammatory responses, Chronic infections, MTT-assay, Heat inactivation, Colon cancer, Breast cancer

## Abstract

A great number of researches over the last years are allocated to know cancer reasons, prevention and treatment strategies. Bacterial infections are one of the promoting factors in cancer development. The present study was carried out to study effects of heat-killed bacteria on cancer cell lines MCF7 and HT-29. To this purpose, four bacterial strains including *Salmonella typhi*, *Staphylococcus epidermidis*, *Escherichia coli *and *Pseudomonas aeruginosa *were assayed. Thermal inactivation method was used to kill the bacteria and preserve the bacterial surface proteins unchangeable. The concentrations of 0.01, 0.1, 0.5 and 1 mg/ml of inactivated bacteria were prepared to evaluate the effects of heat-inactivated bacterial solutions on MCF7 and HT-29 cell lines. MTT assay was used to measure the cell viability of cancer cells treated with different concentration of inactivated bacterial solutions.The MTT assay results after 48 hours showed that the heat-killed bacterial solutions were able to induce the proliferation of both cancer cell lines. In addition, the most cell viability in MCF-7 cell line was seen in samples treated with *S. epidermidis, *while in HT29 cells, the most one was seen in *S. typhi* treated samples. It was concluded that bacterial infections are cancer-deteriorating agents, and any species of bacteria is specific to certain cancerous tissue.

## INTRODUCTION

Cancer is a refractory disease resulted from several changes in cell division-related genes. A great number of researches in recent years are allocated to know different aspects of cancer, including its reasons, prevention and treatment strategies. It is demonstrated that many factors are involved in induction of cancer-leading mutations in the germ line and somatic cells. UV light, X-rays, chemicals, tobacco products, stress and viruses are such of these factors [1-3]. Furthermore, the bacterial infections as promoting factors in cancer development have been assayed for more than two decades. The results showed that the *Helicobacter pylori* infection is one of the first-class carcinogens and is a very important agent in gastric cancer and mucosa-associated lymphoid tissue (MALT) lymphoma [4-7]. In addition to *H. pylori*, the relevance of *Salmonella typhi* to gallbladder cancer, *Streptococcus bovis/gallolyticus* to colorectal carcinoma (CRC) [8-10] and *Chlamydia pneumonia *to lung cancer [11-15] have been distinguished in further investigations. 

In fact, contrary to previous theories that believed bacteria could cause only acute diseases, it is now proved that many bacteria are the cause of chronic infections and diseases, including cancer. It is estimated that over 15% of malignancies worldwide can be attributed to bacterial infections [7] or about 1.2 million cases per year [16]. It is suggested that the relationship between bacteria and carcinogenesis can be both causative and opportunistic [17]. In the causative relation, bacteria induce the inflammatory responses in host body constantly due to the persistence of bacteria itself or bacterial toxins and mediators which are released chronically [18-21]. 

The aim of this study was to analyze the effects of heat-inactivated bacteria on cancer cells in vitro to find whether a killed bacterium could have effects on cancerous cells or vitality is necessary for affecting. In literatures, to inactivate the bacterial cells and preserve their surface structures, different fixation methods has been demonstrated. Inactivation by chemical agents [22], heat [23], sonication [24] and UV irradiations [25, 26] are of these methods. Most commonly used chemical fixatives includes 2.5% glutaraldehyde, 10% formalin, 4% paraformaldehyde, methanol/acetone and ethanol/acetic acid solutions [27]. Although all of these solutions are appropriate in preserving bacterial cell morphology, aldehyde-based solutions are preferred to alcohol ones, because the existence of alcohol results in detaching of the surface ultrastructures (i.e., Pilli and flagella) [27]. Thermal inactivation is one of the most historical and important preservation methods. Microorganisms are more sensitive to wet heat than to dry heat [23]. Damage to the membrane is the main mechanisms of wet heat inactivation [28, 29], while oxidation and protein denaturation is more likely in dry heat inactivation [30]. A non-thermal alternatives to conventional thermal approaches, sonication is mostly used coupled with pressure and/or heat to inactivate the microbes. Spores of bacteria are relatively resistant to this method, thus prolonged periods of ultrasonication would be required [24]. Quantitative and qualitative researches indicate that the amount of resistance to each method is different between gram negative and gram positive bacterial cells. Furthermore, based on images acquired by atomic force microscopy (AFM), the morphology of the cells after fixation is various dependent on utilized method. In this work, based on the purpose of research, the structure of interested bacteria species and advantages and disadvantages of mentioned fixation methods, the wet heat inactivation was chosen.

## MATERIALS AND METHODS

**Preparation of cell lines and bacterial species: **Colorectal adenocarcinoma HT-29 cell line (ATCC HTB-38) was purchased from “National Center for Genetics and Biotechnology of Iran”. Breast cancer MCF-7 cell line (ATCC HTB-22) was supplied by “The Pasteur Institute of Iran”. Four bacterial species including S. typhi (PTCC1609), S. epidermidis (PTCC1436), E. coli (ATCC 25922) and P. aeruginosa (PTCC 1074) were supplied by “The Pasteur Institute of Iran”. 

**Inactivation of bacteria: **To inactivate the bacterial cells and preserve their surface structures, different fixation methods have been demonstrated. Inactivation by chemical agents [47], heat [48], sonication [49] and UV irradiations [50, 51] are of these methods. Most commonly used chemical fixatives include 2.5% glutaraldehyde, 10% formalin, 4% paraformaldehyde, methanol/acetone and ethanol/acetic acid solutions [52]. Although all of these solutions are appropriate in preserving bacterial cell morphology, aldehyde-based solutions are preferred to alcohol ones, because the existence of alcohol results in detachment of the surface ultrastructures (i.e., Pilli and flagella) [52]. Thermal inactivation is one of the most historical and important preservation methods. Microorganisms are more sensitive to wet heat than to dry heat [48]. Damage to the membrane is the main mechanisms of wet heat inactivation [53, 54], while oxidation and protein denaturation are more likely in dry heat inactivation [55]. 

A non-thermal alternatives to conventional thermal approaches, sonication is mostly used coupled with pressure and/or heat to inactivate the microbes. Spores of bacteria are relatively resistant to this method, thus prolonged period of ultrasonication is required [49].

Quantitative and qualitative researches indicate that the amount of resistance to each method is different between gram negative and gram positive bacteria cells. Furthermore, based on images acquired by atomic force microscopy (AFM), the morphology of cells after fixation is various dependent on utilized method. In this work, based on the purpose of research, the structure of interested bacteria species and advantages and disadvantages of mentioned fixation methods, the wet heat inactivation was chosen. 

To prepare the bacterial culture, bacterial species were grown separately in 100 ml of nutrient broth (NB) at 37℃ for 24h with shaking rate of 160 rpm. As mentioned above, in this study, thermal inactivation was used as inactivation method to preserve the bacterial surface proteins unchangeable and to kill the bacteria cells completely. For this purpose, cultured bacteria were precipitated by centrifugation in 4000 ×g for 10 min. After discharging the supernatant, the pellet was washed three times with 2 ml of PBS. Finally, the pellet was resuspended in 1 ml PBS. Inactivation step was applied by placing the obtained solutions in the water bath at 70℃ for 40 min. To insure that all of the bacterial cells were killed, a cultured plate of bacteria was provided and incubated overnight. The solutions of bacteria were dried by freeze-drying. Different concentrations of bacterial solutions including 0.1, 1, 5 and 10 mg per 1 ml PBS were prepared (note that, in MTT assay, the final concentration of solutions in each well will be diluted 10-fold). 

**MTT assay: **MCF-7 and HT-29 cell lines were cultured in RPMI 1640 culture media at 37℃, 5% CO2 and 95% humidity. When the confluency of cells in culture flasks reached 80 percent, cells were detached by trypsin 5% and centrifuged at 500 ×g. The pellet was resuspended in RPMI and aliquoted in 96-well plate, so that approximately 3 × 104 cells were cultured in each well. Then, 20 μl of prepared bacterial solutions were added to wells. The plate was incubated in 37℃, 5% CO2 and 95% humidity for 48 hours. Afterward, 100 μl of culture media was discarded, and 22 μl of MTT solution was added. After 2 hrs incubation, the whole culture media was discarded and 100 μl DMSO was added to each well. The optical density of viable cells was read at 492-630 nm by ELISA reader.

## RESULTS

**Figure 1:**
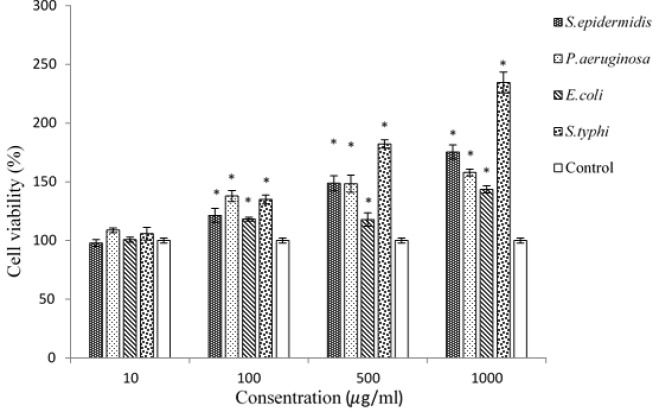
The column graph of MTT assay results from HT-29 cell line treated with four different heat-killed bacterial strains. * Distinguishes the significant difference of samples compared to control

**Figure 2:**
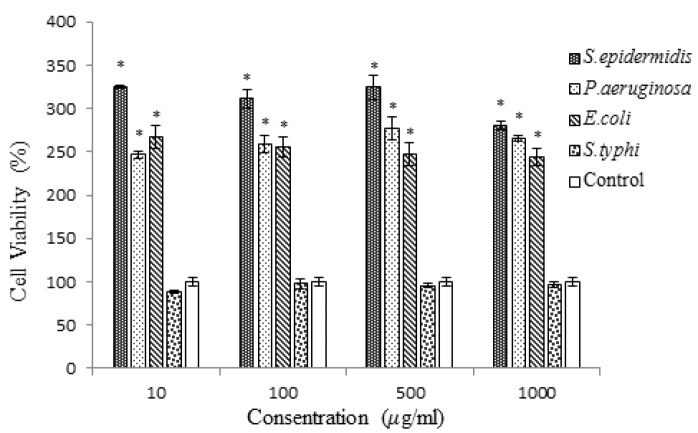
The column graph of MTT assay results from MCF7 cell line treated with four different heat-killed bacterial strains. * Distinguishes the significant difference of samples compared to control

In this research, the MTT assay was performed to evaluate the effects of four different heat-inactivated bacteria strains, introduced as carcinogenic agents, on HT-29 and MCF-7 cell lines. The MTT assay results of HT-29 and MCF7 are shown in Figures 1 and 2, respectively. Furthermore, the outputs of MTT assay were analyzed by SPSS ANOVA v.21 statistical analytical software. P < 0.05 was considered to be statistically significant. According to Fig. 1, cancer-promoting effects on HT-29 cell line were heterogeneous among the four bacterial strains. The highest cancer induction activity was observed by *S. typhi *at the concentration of 1000μg/ml, followed by* S. epidermidis, P.aeruginosa and E.coli. *

*S. typhi *increased the number of cancer cells in a concentration-dependent manner up to 234.54 % compared to negative control. In contrast, the least induction effect obtained by samples treated with *E. coli *solution. The MTT assay showed that among all bacterial strains, along with enhancement of bacterial solutions’ concentrations from 10 to 1000μg/ml, the proliferation of cancer cells and their percentage of cell viability were increased. The comparison of different strains with each other showed that the increasing effect of *P. aeruginosa *was more than samples treated with *E. coli* in all concentration. Furthermore, inactivated *S. typhi *induced the proliferation of cancer cells more than inactivated *E. coli* and *S. epidermidis* in all concentrations. In Fig. 2, the MTT assay results of MCF-7 cell line are shown. Three species including *S. epidermidis, P. aeruginosa and E. coli *had significant induction effect on cancer cells, while *S. typhi *had no effect on cancer cells. The most increasing activity was observed by *S. epidermidis *at all concentrations. *S. epidermidis *effect showed ascending trend by increasing the concentration of the bacterial solution from 10 to 500μg/ml, but at the concentration of 1000 μg/ml, a slight reduction on cell viability of cancer cells was observed. The most cell viability of MCF-7 cells, 324.24%, was obtained by samples treated with 500μg/ml of *S. epidermidis.*

Also, *P. aeruginosa *had significant induction effect on cells growth at all concentrations. The E. coli increased the proliferation of cancer cells at all concentrations. Its effect was dose-dependent with the slight negative slope.

## DISCUSSION

In this work, the relationship of bacteria with cancer cells was assayed via in vitro experimental approaches. For this purpose, the effects of heat-inactivated of four different bacteria on breast and colon cancer cell lines were studied by MTT assay. Because of toxic effects of whole living bacterium on eukaryotic cells in MTT test, the bacterial solutions have to be inactivated. Consequently, the thermal inactivation method was selected to inactivate the internal metabolism of bacteria and to conserve their surface structures. The reasons have been explained in the following. On the one hand, it is distinguished that among inactivation methods for bacterial cells, inactivation with formaldehyde, paraformaldehyde and heat are better in preservation of three- dimensional structures of surface proteins [27, 31]. On the other hand, paraformaldehyde and formaldehyde are toxic to eukaryotic cells; therefore, remaining traces of them in bacterial solutions due to incomplete washing step can be problematic in obtaining true results. During recent years, a lot of researches allocated to study challenging incorporation mechanisms of bacterial cells in cancer. Some researchers believe that their relationship is more opportunistic rather than causative [32-36]. In tumor tissues due to some appropriate conditions including the existence of adequate bacterial nutrients and providing a refuge for bacteria to evade the immune system clearance [37-40], bacteria tend to enter and accumulate in tumorous tissues based on opportunism. On the contrary, it is believed by some researchers that bacteria are the causative factors in the cancer-promoting process [11, 36, 41-45], because they induce the chronic inflammatory response in host cells. Furthermore, it is investigated that microbial flora of cancerous tissues is not the same as one in normal tissue. Some researches demonstrate that microbial flora of breast cancer patients is different from normal persons, such that bacteria belonging to three genera, *Enterobacter*, *Pseudomonas* and *Staphylococcus*, were extracted from breast cancer patients [46, 47]. Also, Cantwell and et al. extracted *S. epidermidis* from breast cancer tissues [48].

In the present research, the results of MTT assay of *S. epidermidis, P. aeruginosa *and *S. typhi *on MCF-7 and HT-29 cell lines showed that presence of these bacteria in tumorous tissues, especially in breast cancer tissues, even after becoming cancerous, can exacerbate the growth of cancer cells. In other words, the taken changes in the bacterial flora of the tissues will end in favor of cancerous cells.

In the MTT assay, the MCF-7 cell line was more influenced by bacterial treatment than HT-29 cells. The most cell growth and cell viability in MCF-7 cell line were seen in samples treated with *S. epidermidis, P. aeruginosa* and* E. coli, *respectively, and no significant cell growth was seen in *S. typhi* treated wells (Figure 2); however, in HT-29 cell line the most cell viability was seen in samples treated with *S. typhi *(Figure 1). Presumably, it is because of specific interactions between bacterial species and eukaryotic cell surface proteins. It has been indicated that S. typhi recognizes a specific glycosylation pattern, GlcNAc*β*1-4GlcNAc*β*-N/Gly, on colon cancer cells and makes a connection with these cells [49]. Because the mentioned glycosylation pattern is not detected on breast cancer cells [50], *S. typhi* cannot make a junction with these cancer cell receptors. It is supposed that this weak connection is the reason of having no significant effect of *S. typhi* solutions on MCF-7 cells in this research.
